# Insights on the Synthesis of Al-MCM-41 with Optimized Si/Al Ratio for High-Performance Antibiotic Adsorption

**DOI:** 10.1021/acsomega.3c07119

**Published:** 2023-12-09

**Authors:** Vinicius M. S. Macedo, Eliezer L. Gomes, Juan C. Moreno-Piraján, Liliana Giraldo, Laura P. Tovar, Sarah I. P. M. N. Alves, Luís A. M. Ruotolo, Romilda Fernandez-Felisbino

**Affiliations:** † Department of Chemistry, Federal University of São Paulo, Diadema, SP 09972-270, Brazil; ‡ Department of Chemical Engineering, Federal University of São Paulo, Diadema, SP 09972-270, Brazil; § Facultad de Ciencias, Universidad de los Andes, Carrera 30 No. 45-03, Bogotá 01, Colombia; ∥ Department of Physics, Federal University of São Paulo, Diadema, SP 09972-270, Brazil; ⊥ Department of Chemical Engineering, Federal University of São Carlos, São Carlos, SP 13565-905, Brazil; ▽ Facultad de Ciencias, Universidad Nacional de Colombia, Carrera 30 No. 45-03, Bogotá 01, Colombia

## Abstract

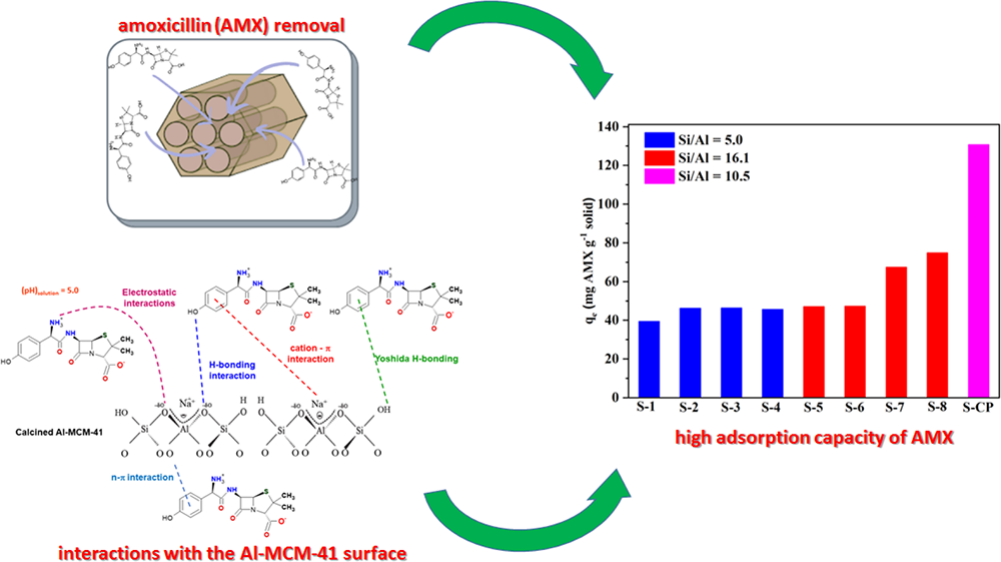

Studies indicate that approximately two-thirds of the rivers of the world are contaminated by pharmaceutical compounds, especially antibiotics and hormones. Data reported by the World Health Organization (WHO, 2015) revealed an increase of 65% in antibiotic consumption between 2000 and 2015, with a worldwide increase of 200% expected up to 2030. Environmental contamination by antibiotics and their metabolites can cause the alteration of bacterial genes, leading to the generation of superbacteria. In this work, adsorption was explored as a strategy to mitigate antibiotic contamination, proposing the use of the Al-MCM-41 mesoporous material as an efficient and high-capacity adsorbent. Evaluation of the influence of the synthesis parameters enabled understanding of the main variables affecting the adsorption capacity of Al-MCM-41 for the removal of a typical antibiotic, amoxicillin (AMX). It was found that the adsorbent composition and specific surface area were the main factors that should be optimized in order to obtain the highest AMX removal capacity. Using statistical tools, the best Si/Al ratio in Al-MCM-41 was found to be 10.5, providing an excellent AMX uptake of 132.2 mg per gram of adsorbent. The Si/Al ratio was the most significant factor affecting the adsorption. The cation−π interactions increased with an increase of the Al content, while the interactions involving silanols (Yoshida H-bonding and dipole–dipole hydrogen bridges) decreased.

## 1 Introduction

Water, one of the most important natural resources, is frequently contaminated by a variety of emerging micropollutants; therefore, there is an urgent need to manage the impacts caused by the various forms of contamination. Research indicates that around two-thirds of the rivers worldwide are contaminated by pharmaceutical compounds, notably antibiotics and hormones. Data from 76 countries for the period from 2000 to 2015, reported by the World Health Organization (WHO), show that there was a 65% increase in antibiotic consumption, from 21.1 to 34.8 billion defined daily doses (DDDs).^1^ The projected worldwide increase in antibiotic consumption up to 2030 indicates that consumption will be approximately 200% higher than in 2015. As reported by Homem and Santos, the contamination of aquatic systems by antibiotics is mainly due to anthropic activities such as discharges of domestic sewage and hospital wastes.^2^ Additionally, residues of antibiotics used in agriculture, aquaculture, poultry farming, and pet health treatments contribute to the contamination. Industrial sources include effluents from pharmaceutical industries.^3^ The presence of these micropollutants has been detected in surface waters, groundwater, effluent treatment plants, and supply waters.^2,3^

Amoxicillin (AMX), one of the most consumed antibiotics worldwide, is a β-lactam compound belonging to the penicillin class. Although AMX presents high bioavailability, only about 15% is effectively metabolized in the body, while the rest is excreted in urine and feces, which are discharged in effluents.^4,5^ The release of antibiotics into the environment leads to frequent contact with bacteria, making them more resistant, with dissemination of antibiotic and antimicrobial resistance (AMR).^6^ In addition to the serious environmental impacts, major public health problems can be expected due to the inefficacy of conventional antibiotics in combating superbacteria. According to research carried out by the European Centre for Disease Prevention and Control (ECDC), around 33,000 people die every year due to aggravations caused by superbacteria.^6,7^ In light of this, increasing interest of the scientific community has been directed toward the development of methodologies for the removal of antibiotics from aqueous systems.

There are several technologies that can be employed to remove pharmaceutical compounds from aquatic media, such as biological processes,^8^ advanced oxidation processes,^9^ ozonation,^10^ Fenton/photo-Fenton and semiconductor photocatalysis,^11,12^ membranes,^13^ and adsorption processes.^5,14^ It is important to note that some of these methods are costly or have a complex operation, making them impracticable on a large scale. However, adsorption stands out due to its easy operation, low cost, and high efficiency in removing contaminants present at low concentrations.^14^ These aspects have made adsorption the most efficient approach reported in the literature for the removal of contaminants such as pharmaceuticals.^3,5,15^ There is now strong interest in identifying new adsorbents that offer enhanced adsorption performance.

Considering that adsorption is a surface phenomenon, it is expected that higher specific surface area (SSA) should increase the efficiency of contaminant uptake.^16^ Promising adsorbents that have been described include activated charcoal/biochar,^3,4,17,18^ clay,^5^ and zeolites.^19^ Specifically for the removal of antibiotics, hierarchically multiporous carbon nanotubes,^20^ metal oxides/graphene,^21^ and functionalized mesoporous silica are examples of adsorbents used for this purpose,^22−24^ with a focus on the synthesis of materials with high SSA in order to achieve high uptake capacity. However, although high SSA is desirable, antibiotic uptake is also influenced by the number of adsorption sites and the ability of the contaminant to access these sites. Furthermore, interaction with a solid surface possessing a framework composition can also enhance antibiotic adsorption. Most of the reported studies concerning the adsorption of antibiotics have employed functionalized mesoporous silica, obtained using postsynthesis treatments.^19−24^ However, until now, no reports were found regarding the adsorption of AMX using Al-MCM-41 obtained by using isomorphic substitution during the hydrothermal synthesis process.

Considering these aspects, this work explores the use of Al-MCM-41 silica as an efficient and high-capacity adsorbent for the removal of amoxicillin, selected as a typical antibiotic for studying adsorption performance. A systematic investigation was performed to optimize the adsorption capacity and clarify the effects of synthesis parameters, including the Si/Al ratio on the properties of Al-MCM-41, such as specific surface area.

## 2 Experimental Section

### 2.1 Synthesis of Al-MCM-41

A factorial design of the type 2^k^ + 3CP was employed to investigate the effects of the factors (*k*) Si/Al (*X*_1_), temperature (*X*_2_), and time (*X*_3_). The levels of the coded variables are listed in Table 1. Three central points (CP) were added to estimate the variability and curvature, resulting in a set of 11 experiments (Table 2). The Si/Al ratio or Al content (***x***) was calculated by considering the molar compositions, according to eq 1.
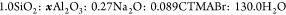
1

**Table 1:** Levels of the Coded Variables of the Factorial Design

		**level**		
**factors**		**-1**	**0**	**+1**
Si/Al ratio	Si/Al	5.0	10.5	16.1
temperature (°C)	T	100	125	150
time (h)	t	8	10	12

**Table 2:** Lattice Parameters of the Samples and the Synthesis Conditions

**run**	**Si/Al**	***T* (°C)**	***t* (h)**	**2θ (*d***_**100**_**)**	**θ (*d***_**100**_**)**	**sinθ**	***d***_**100**_^a^	***a***_**0**_^b^
1	5.0	100	8	2.2	1.1	0.019	40.7	47.0
2	5.0	150	8					
3	5.0	100	12	2.2	1.1	0.019	40.5	46.8
4	5.0	150	12					
5	16.1	100	8	2.3	1.1	0.020	38.8	44.8
6	16.1	150	8	2.2	1.1	0.019	40.1	46.3
7	16.1	100	12	2.3	1.2	0.020	37.6	43.4
8	16.1	150	12	2.2	1.1	0.019	39.6	45.7
9	10.5	125	10	2.2	1.1	0.019	40.8	47.1
10	10.5	125	10	2.2	1.1	0.019	40.8	47.1
11	10.5	125	10	2.2	1.1	0.019	40.7	47.0

a λ = 2*d*_100_sin θ.

b *a*_0_ = (2/√3) *d*_100_.

The mesoporous Al-MCM-41 silica was synthesized according to a modification of the procedure previously described in the literature.^25,26^ Briefly, the synthesis consisted of the dissolution of CTMABr (Riedel-de Han) and the Si source (TEOS, Sigma-Aldrich) in an alkali solution (the concentration of NaOH is given by eq 1), under stirring at room temperature (30 min), resulting in a turbid solution into which was poured an aqueous solution containing the Al source (NaAlO_2_, Synth). The resulting solution was first stirred at room temperature (120 min), then at 80 °C (20 min), and again at room temperature (4 h). The gel obtained (with the molar composition shown in eq 1) was poured into a Teflon vessel, which was placed in a stainless-steel autoclave and left in an oven for hydrothermal treatment by using the temperatures and times stipulated in the experimental design (Table 1). After filtration, the solids were washed with water until neutral pH, followed by drying at 120 °C for 12 h. In order to remove the organic residues, the solids were calcined for 5 h at 550 °C, with heating at 2 °C min^–1^, under an oxidizing atmosphere.

### 2.2 Characterizations

The synthesized and calcined Al-MCM-41 solids were characterized before being applied in adsorption experiments. Diffractograms of the solids were acquired using an X-ray diffractometer (model D8 Advance, Bruker) operating with Cu Kα radiation (λ = 1.54 Å), in the 2θ range from 1 to 10°, with scanning at a rate of 0.2° min^–1^. The interplanar distance, *d*_100_, and the lattice parameter of the mesoporous hexagonal arrangement, *a*_0_, were calculated from the diffractograms.

Nitrogen adsorption/desorption isotherms were obtained using liquid N_2_ (at 77 K), in the relative pressure range from 0.06 to 0.97. The samples were previously degassed for 3 h at 200 °C. The analyses were performed by using a NOVA 1200 system (Quantachrome Instruments), with the results recorded by using NOVAWin 2.0 software.

Infrared spectra were acquired using a spectrometer (model IR Prestige-21, Shimadzu) operated in transmittance mode, in the range 4000–400 cm^–1^, with a resolution of 400 cm^–1^. Before the analyses, the solids were dried at 100 °C for 12 h and were then kept in a desiccator with humidity control. For analysis, the samples were diluted at 0.1% in KBr and pressed into tablet form.

Thermogravimetric analyses were carried out using a thermobalance (model DTG-60H, Shimadzu), in the temperature range from 25 to 600 °C, with heating at a rate of 10 °C min^–1^, under an oxidizing atmosphere of air at a flow rate of 50 mL min^–1^.

### 2.3 Amoxicillin Adsorption

Batch AMX adsorption assays were carried out in triplicate in phosphate solution (KH_2_PO_4_) at 25 °C and pH 5.0 (adjusted using 1 M HCl solution), in order to ensure the isoelectric point of the AMX molecule. The adsorbent (2.0 mg) was placed in solutions (1.0 mL) containing AMX at concentrations varying from 40 to 220 mg L^–1^, under agitation using a shaker at 250 rpm, until reaching equilibrium. The solid was then filtered using a 0.22 μm membrane (Millipore). Measurement of the adsorption kinetics was performed using an initial AMX concentration of 200 mg L^–1^ and contact times from 0 to 48 h. Adsorption isotherms were obtained by using a 24 h equilibrium time and initial AMX concentrations of 220, 200, 180, 160, 120, 80, 60, 50, and 40 mg L^–1^.

The adsorption performance was evaluated in terms of the AMX concentration in the solid phase at equilibrium (*q*_e_, mg g^–1^ of adsorbent), determined from the mass balance (eq 2) and the AMX removal efficiency (eq 3). The initial (*C*_0_) and equilibrium (*C*_e_) concentrations of AMX in the liquid phase were determined by UV–vis spectrophotometry (model HP 8453, Hewlett–Packard), measuring the absorbance at 271 nm using a previously constructed AMX calibration curve. The determinations were made in triplicate.
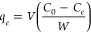
2
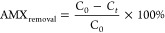
3

## 3 Results and Discussion

### 3.1 Characterizations of the As-Synthesized Adsorbents

**Figure 1:**
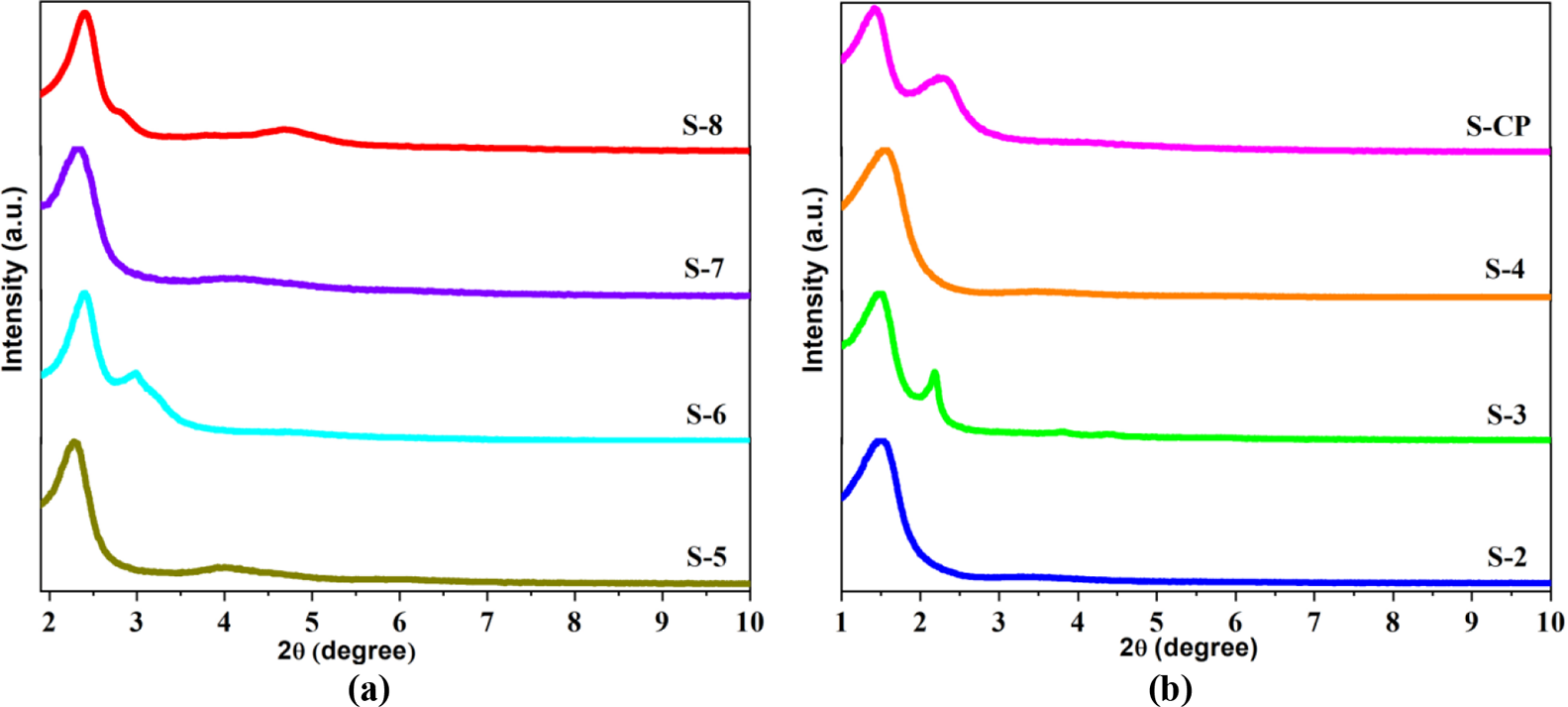
Diffractograms of the calcined Al-MCM-41 samples obtained using Si/Al ratios of (a) 16.1 and (b) 5.0 and 10.5 (S-CP).

The X-ray diffractograms of the set of solids obtained using Si/Al = 16.1 (S-5, S-6, S-7, and S-8) are presented in Figure 1a. The characteristic (100), (110), and (200) diffraction peaks confirmed the formation of the hexagonal Al-MCM-41 silica phase. The solids synthesized using the Si/Al ratio of 16.1 presented a greater degree of organization of the hexagonal structure compared to those obtained using other Si/Al ratios. Increase of the aluminum content in the gel (Si/Al = 5.0 and T = 150 °C) led to the formation of solids (S-2 and S-4) without development of the hexagonal phase (Figure 1b). The diffractograms for samples S-1 and S-3 (Si/Al = 5.0 and T = 100 °C) showed the characteristic peaks of the hexagonal structure but with lower intensity, indicative of poorer structural organization, when compared to the solids obtained using Si/Al = 16.1 (Figure 1a).

The diffractograms shown in Figure 1b for the samples prepared using Si/Al = 5.0 presented wider and lower intensity peaks, similar to those obtained by Cesteros and Haller for Al-MCM-41 with high Al content.^27^ The incorporation of more aluminum into the silica framework led to greater distortion in the ordering of the hexagonal phase. During the thermal treatment, the Al species in solution are preferentially inserted in the silica framework at the beginning of the synthesis; therefore, the higher the Al content, the more difficult it is to obtain an ordered mesophase. Under this condition, the formation of disordered mesopores occurs, with concentration of structural Al on the mesopore surface during solidification of the hexagonal phase.^28^

The diffractogram for the S-CP sample (Figure 1b), synthesized in the central point experiments (S-9, S-10, and S-11), was characteristic of the hexagonal phase with a high degree of organization.^29^ The interplanar distances obtained for the (100) plane, *d*_100_, and the lattice parameters, *a*_0_, of the S-9, S-10, and S-11 solids (Table 2) presented a difference of less than 0.5%, demonstrating that the Al-MCM-41 synthesis was reproducible.

**Figure 2:**
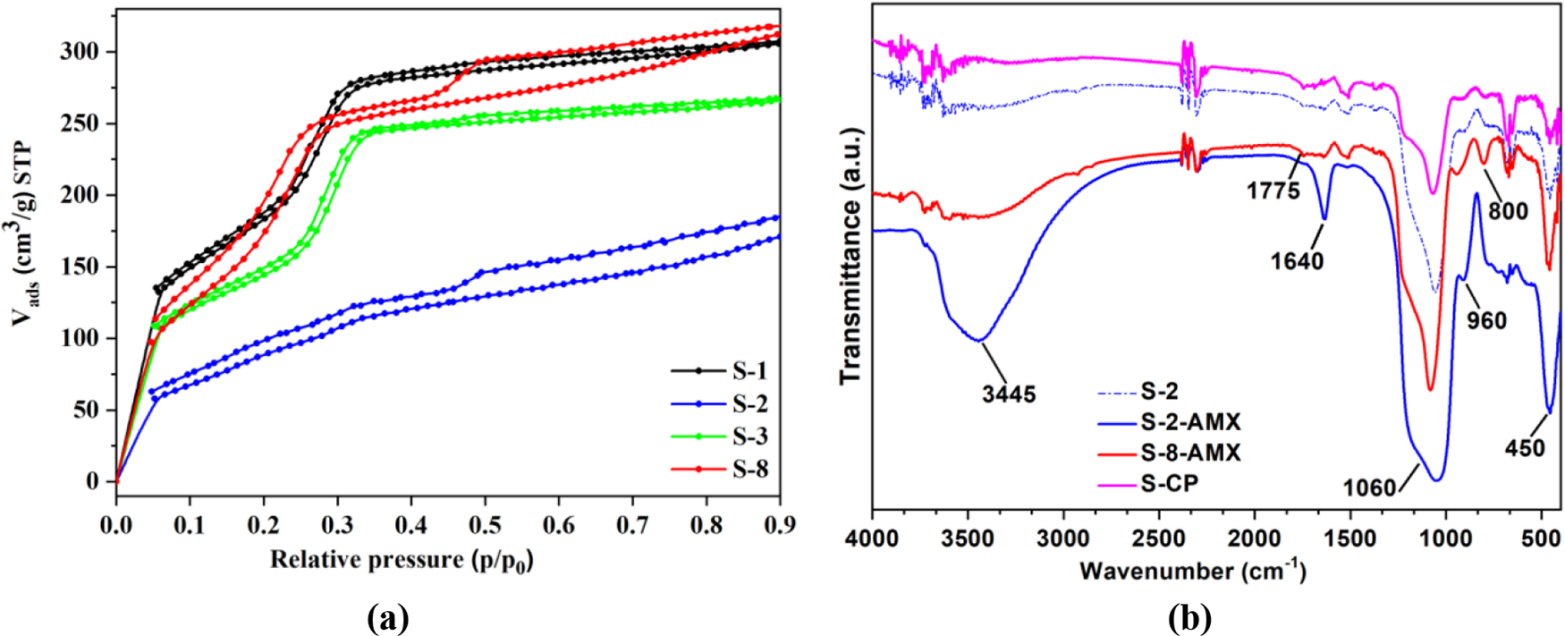
(a) N_2_ adsorption–desorption isotherms for samples S-1, S-2, S-3, and S-8. Liquid N_2_ (at 77 K), in the relative pressure range from 0.06 to 0.97; (b) FTIR absorption spectra for samples S-2, S-9, S-8-AMX, and S-2-AMX.

Figure 2a shows the N_2_ adsorption–desorption isotherms for samples S-1, S-2, S-3, and S-8. The isotherms were typical of type IV, with hysteresis at *P*/*P*_0_ = 0.45, which could be ascribed to capillary condensation in uniform mesopores. The isotherm for sample S-8 (SSA of 840 m^2^ g^–1^) showed stepped desorption, indicative of mesopore heterogeneity.^30^ The isotherm for sample S-2 revealed the formation of mesopores and a smaller specific area (129 m^2^ g^–1^), although mesopore ordering was not evidenced in the X-ray diffractogram (Figure 1b), considering the characteristic peaks of the hexagonal structure.

Figure 2b shows the FTIR absorption spectra for samples S-CP, S-2, and S-2-AMX (with adsorbed amoxicillin). Absorption peaks at 1,060 and 800 cm^–1^ could be attributed to asymmetric and symmetric stretching vibrations, respectively, of the bonds of the Si–O–Si groups. The spectra also showed a peak at 450 cm^–1^, due to deformation of the O–Si–O groups, and a peak at 960 cm^–1^, attributed to stretching of the Si–OH groups.^29,31^ When a hexagonal phase is formed in MCM-41, bands in the range 960–970 cm^–1^ are assigned to Si-OH vibrations, but when metals are incorporated, the intensity of these bands increases, confirming the incorporation of the heteroatom into the framework.^31^ The spectrum for S-2-AMX showed the presence of other peaks at 3,455, 1,775, and 1,640 cm^–1^ assigned to stretching vibrations of free amino groups and −CH, −CH_2_ and CH_3_ groups, and −OH bending corresponding to the functional groups of amoxicillin, which confirmed the adsorption of AMX.^5,32^

**Figure 3:**
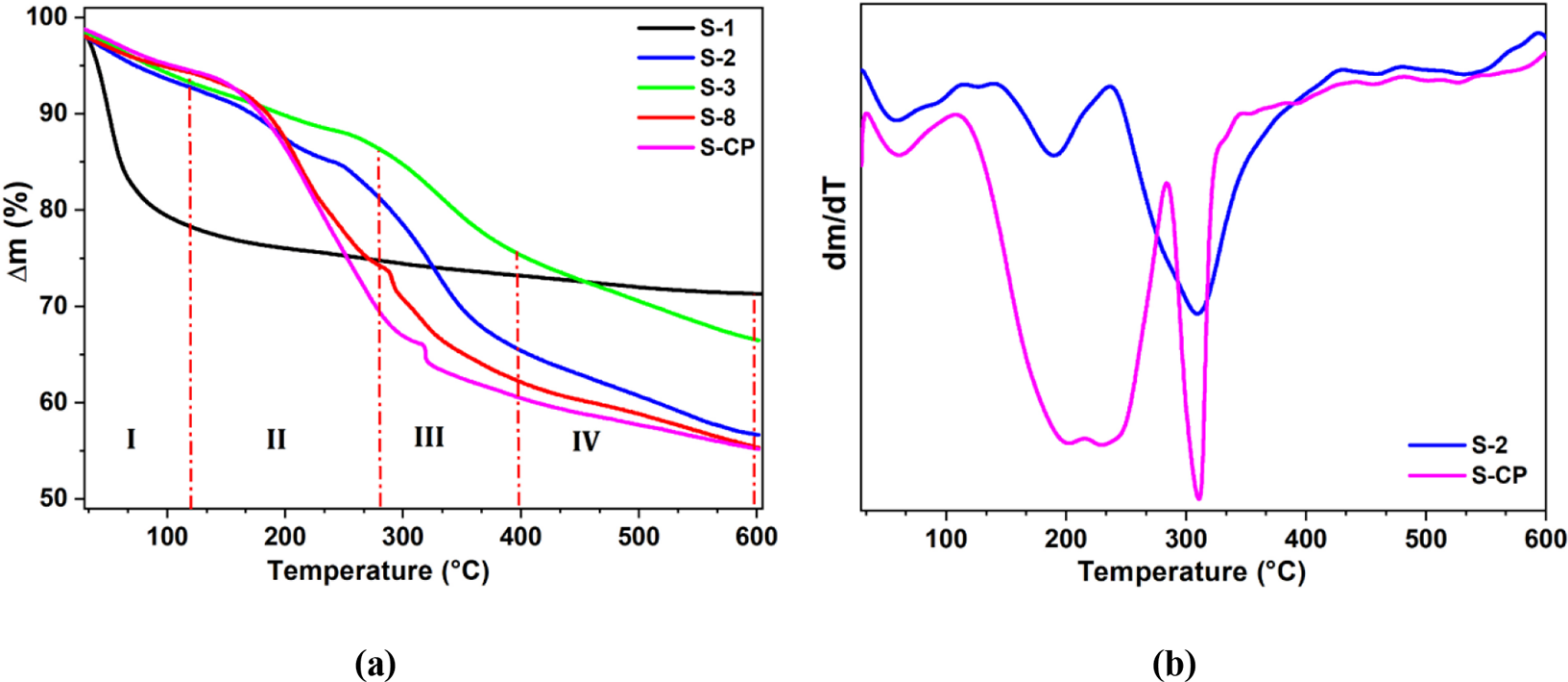
TG (a) and DTG (b) curves for the synthesized samples.

The TG curves (Figure 3a) revealed total mass losses in the range of 30–45%, with greater thermal stability shown by the solids with higher aluminum content. Table 3 shows the mass loss results, where the first mass loss could be explained by the release of water from the silica. The solids with higher Si/Al ratios presented lower mass losses, since a higher Al content in the structure made the solid less hydrophobic.^27^ The mass loss in the range 120–180 °C was due to decomposition of the surfactant (the SiO^–^CTA^+^ bond is weak in the as-synthesized sample). In the range 280–400 °C, the mass loss could be attributed to breakdown of the hydrocarbon chains, while mass loss between 400 and 600 °C could be explained by combustion of the surfactant and loss of water associated with the condensation of the silanol groups.^33^

**Table 3:** Mass losses of the synthesized solids in different temperature ranges (25 to 600 °C, at a rate of 10 °C min^–1^, air at a flow rate of 50 mL min^–1^).

	**Δm (%)**				
**sample**	**25–120 °C**	**120–280 °C**	**280–400 °C**	**400–600 °C**	**Δm_total_(%)**
S-1	22.0	3.40	1.30	1.90	28.60
S-2	6.93	12.27	15.3	8.80	43.30
S-3	6.32	7.67	10.62	8.84	33.45
S-8	5.24	21.06	11.35	6.72	44.37
S-CP	5.58	23.32	8.84	6.85	44.59

The solids synthesized using Si/Al = 16.1 presented a higher mass loss related to the release of the surfactant occluded in the pores compared to the solids obtained using Si/Al = 5 (Table 3).

As shown in Figure 3b, sample S-2 presented higher mass loss in the temperature range 280–400 °C, compared to sample S-CP, reflecting the higher Al content in the mesoporous structure of sample S-2. In this case, the interaction between the surfactant and the Al species was stronger than that between Al and the silanol groups, which was confirmed by the mass loss at higher temperatures.^34^

### 3.2 Adsorption Kinetics and Equilibrium

**Figure 4:**
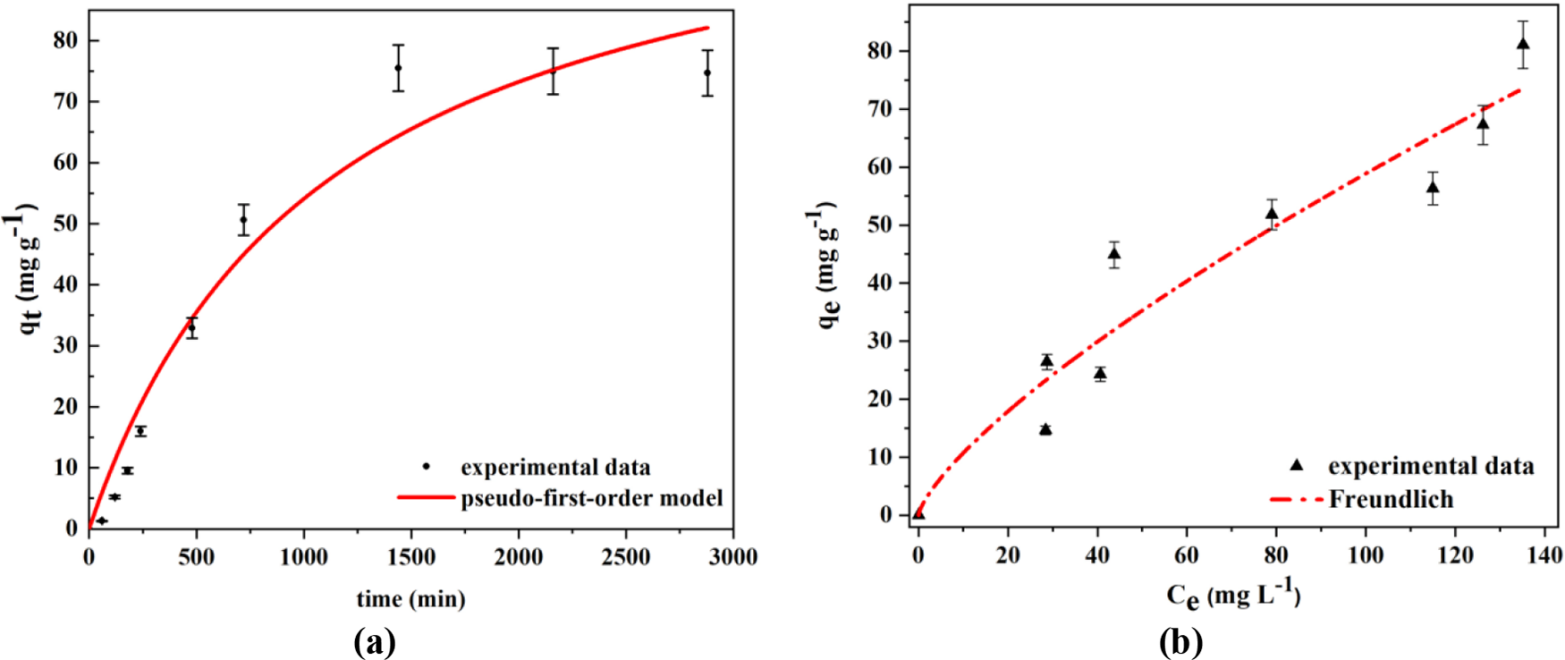
(a) AMX uptake on sample S-8 (Si/Al = 16.1), as a function of contact time (*C*_0_ = 200 mg AMX L^–1^); (b) adsorption isotherm for the S-CP sample. Adsorption conditions: pH 5.0 and 25 °C.

Figure 4a shows the effect of the contact time on AMX adsorption onto sample S-8, which had the highest SSA (840 m^2^ g^–1^). The adsorption was performed at the isoelectric point (pH 5.0), in order to neutralize the influence of the charges of functional groups during the process. Accordingly, the kinetics followed a nonlinear pseudo-first order model (*q*_*t*_ = *q*_*e*_(1 – e^–*k*1*t*^), *k*_1_ = 1.20 ± 0.19 min^–1^, and *R*^2^ = 0.977), with a maximum adsorption capacity of 82.5 mg g^–1^ at equilibrium, reached after 24 h. This behavior observed for the adsorption of AMX on Al-MCM-41 was different from that reported for adsorbents such as activated charcoal/biochar,^4,35^ multiwalled carbon nanotubes,^35^ and organobentonite clay,^36^ which presented pseudo-second order kinetics due to the fast uptake at the beginning of the process, caused by the large number of free adsorption sites. Therefore, the kinetic behavior suggested that the adsorption mechanism was different using Al-MCM-41.^36^

The equilibrium thermodynamics of the adsorption using Al-MCM-41 was evaluated by using the isotherm for AMX uptake on the S-CP sample (Figure 4b). The best fit to the data was obtained with the nonlinear Freundlich model (*q_e_* = *K*_f_*C*_*e*_^1/*n*^).

The Freundlich constants referring to the adsorption capacity and affinity coefficient were *K_F_* = 0.0023 mg g^–1^ and *n* = 0.383 (L mg^–1^)^1/n^, respectively, with *R*^2^ = 0.943. The high 1/*n* value (2.61) of the Freundlich model indicated low affinity of AMX for the Al-MCM-41, due to surface heterogeneity,^29,34^ as observed elsewhere for AMX adsorption on activated carbon.^37^

All the samples displayed similar kinetics and isotherm trends, with no statistically significant influence of the variables studied on the pseudo-first order constants. On the other hand, the values of %*R* and *q*_*e*_ were sensitive to the properties of the adsorbents obtained using different synthesis conditions. The values of %*R* and *q*_*e*_ (means of triplicates) are shown in Table 4 for the different conditions employed in the factorial design.

**Table 4:** AMX Removal Percentages (%*R*) and Adsorption Capacities At Equilibrium (*q*_*e*_)

**run**	**Si/Al**	***T* (°C)**	***t* (h)**	**%*R***	*q*_*e*_**(mg g**^**–1**^**)**
1	5.0	100	8	15.9	39.5
2	5.0	150	8	18.6	46.2
3	5.0	100	12	18.5	46.4
4	5.0	150	12	18.1	45.6
5	16.1	100	8	19.1	47.1
6	16.1	150	8	18.9	47.3
7	16.1	100	12	27.4	67.5
8	16.1	150	12	29.9	74.9
9	10.5	125	10	33.2	124.4
10	10.5	125	10	34.2	130.8
11	10.5	125	10	34.4	130.9

**Figure 5:**
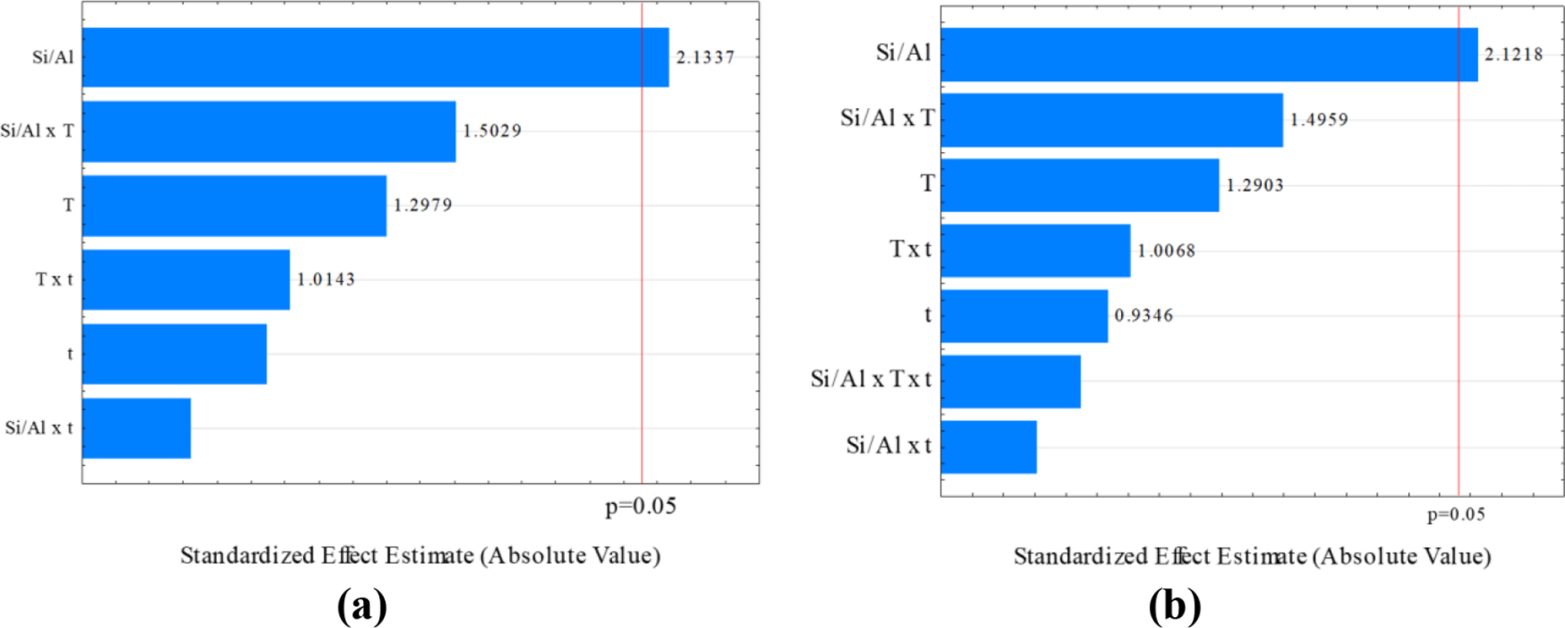
Pareto charts of the standardized effects (*p* = 0.05) for (a) %*R* and (b) *q*_*e*_.

As a first attempt to understand the role of the synthesis parameters in the adsorption of AMX by Al-MCM-41, a factorial design was applied for a systematic study of the effects of the synthesis gel Si/Al ratio, temperature, and reaction time.^38^ The as-obtained adsorbents were evaluated in terms of AMX removal (%*R*) and the adsorption capacity at equilibrium (*q_e_*). The synthesis variables were statistically analyzed considering their individual and interaction effects. The variance analysis adopted a 5% significance level (*p* < 0.05), indicated by the red lines in the Pareto charts of the effects (Figure 5). For the levels of time and temperature studied, only the individual effect of the Si:Al ratio significantly influenced the adsorption process. Hence, for the same Si/Al ratio, the effects of time and temperature on the Al-MCM-41 adsorption properties were solely explained by the variance. Application of analysis of variance (ANOVA) showed that the *F*-values (for the model and the error) were higher than the *F*-distribution values for the factors analyzed, indicating that the statistical model was suitable for describing the response variables at 5% significance.

### 3.3 Influence of synthesis parameters on adsorptive properties

**Figure 6:**
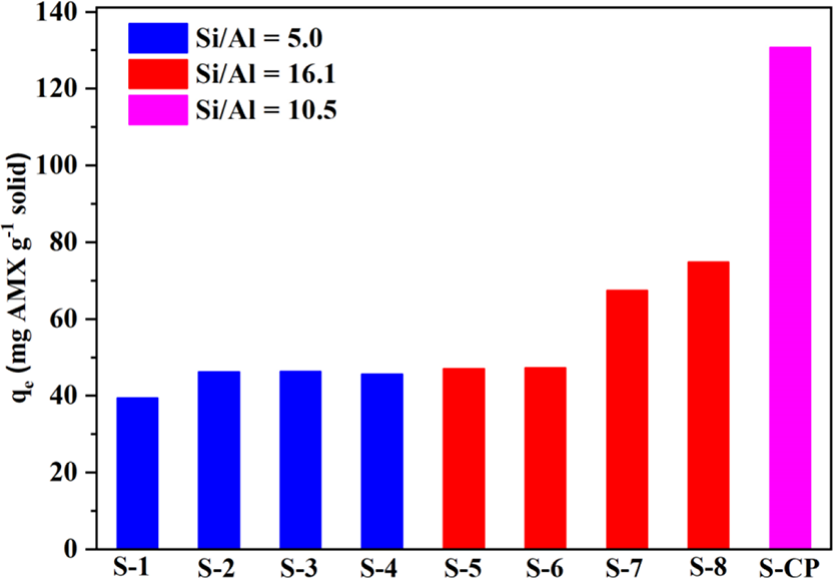
AMX adsorption capacities at equilibrium (*C*_0_ = 200 mg L^–1^, pH 5.0, and 25 °C).

The AMX adsorption capacities (*q*_*e*_) of the Al-MCM-41 samples varied from 40 to 132 mg g^–1^ (Figure 6), indicating the influence of the synthesis conditions on the properties of the adsorbent. The best performance was observed for the S-CP sample (132 mg g^–1^), synthesized according to the conditions of the central points (S-9, S-10, and S-11) of the factorial design. It should be highlighted that this adsorption capacity far exceeded the values reported in the literature for other adsorbents used for antibiotic removal, such as multiwalled carbon nanotubes/iron nanoparticles (23.4 mg g^–1^),^37,39,40^ mesoporous calcium carbonate (13.5 mg g^–1^),^41^ and MCM-41/CTA composite (55 mg g^–1^).^42^

Interestingly, sample S-8 (Si/Al = 16.1) presented higher SSA (840 m^2^ g^–1^) than sample S-2 (Si/Al = 5.0), for which the value was only 129 m^2^ g^–1^, despite its high aluminum content, confirmed by the mass loss at 280–400 °C (Figure 3b), and low degree of order of the hexagonal arrangement, confirmed by the absence of the characteristic peaks in the X-ray diffractogram (Figure 1b). These results suggested a tradeoff between the Si/Al ratio and the SSA, which influenced the adsorption properties, since the value of *q*_*e*_ for S-8 (80.0 mg g^–1^) was 50% higher than observed for S-2 (46.2 mg g^–1^) (Figure 6). In principle, this could be mainly ascribed to the much lower surface area of S-2. However, this was not the only property affecting the adsorption capacity since the SSA was 6.5-fold higher for S-8, compared to S-2, while *q*_*e*_ was only 1.7-fold lower for S-2.

It is possible that the presence of Al species in the mesoporous structure of S-2 could have significantly contributed to AMX removal, despite the less organized hexagonal arrangement (Figure 1) promoted by the higher Al content in the synthesis medium, leading to the incorporation of Al^3+^, instead of Si^4+^. The difference in the atomic radii of Al and Si (53 and 40 pm, respectively) would have created distortions during formation of the mesophase, leading to greater availability of Al^3+^ species on the mesopore surface.^28^ These species could then contribute to the AMX adsorption, providing an explanation for the fact that the adsorption capacity of S-2 was higher than expected if only SSA was considered.

### 3.4 Mechanisms for Adsorption of AMX on Al-MCM-41

**Figure 7:**
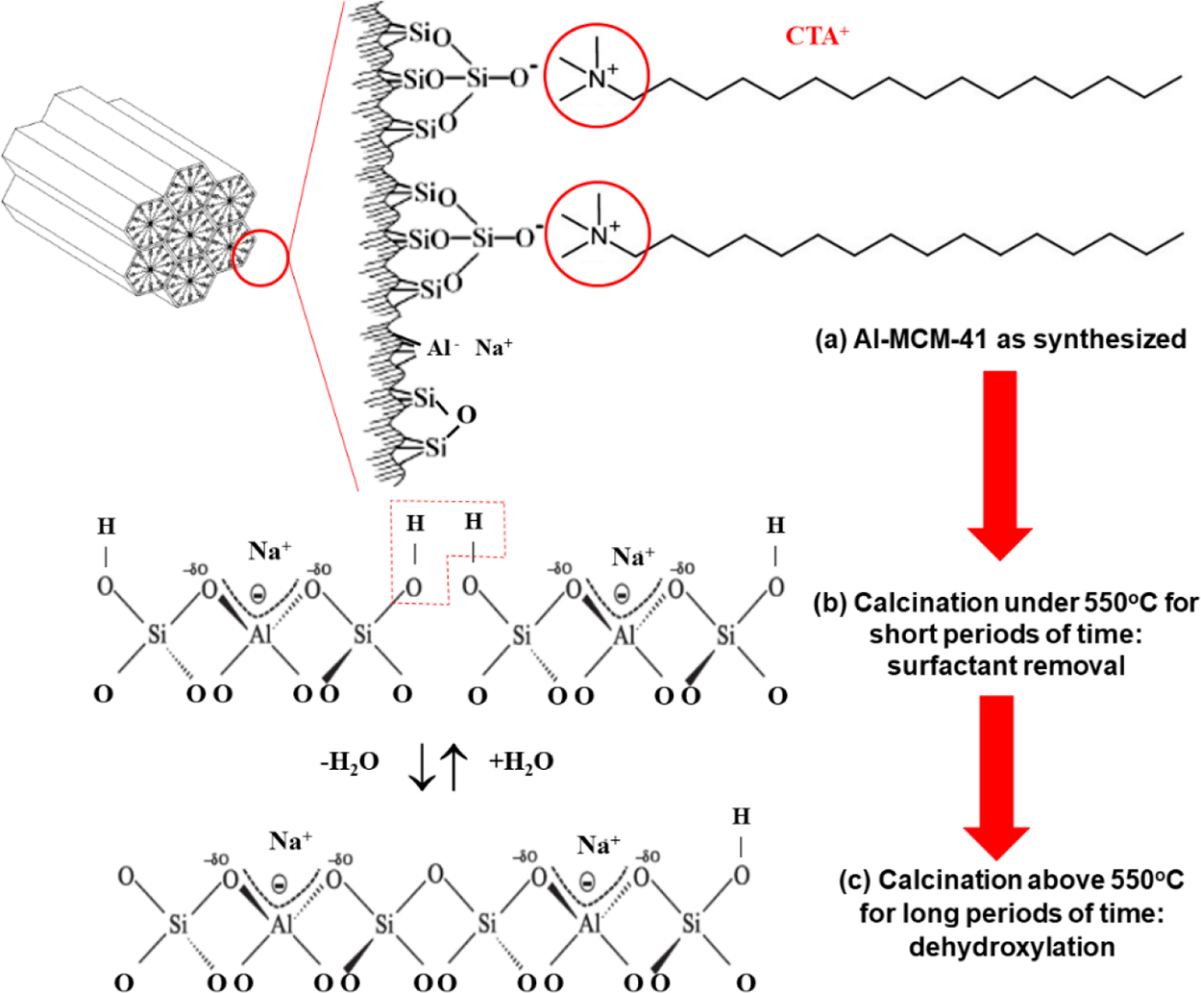
Schematic illustration of the Al-MCM-41 aluminosilicate framework. (Adapted with permission from Naik *et al*., 2010. Copyright 2023 Elsevier).

Figure 7 shows a schematic illustration of the probable surface of Al-MCM-41 after controlled calcination at 550 °C in an oxidizing atmosphere. Removal of the surfactant resulted in structural alteration, with reorganization of the chemical bonds leading to partial contraction of the walls, due to new [Si-O-Si] or [Si-O-Al] bonds, consequently slightly decreasing the diameter of the mesopores.^26,43,44^ Therefore, the material obtained had an aluminosilicate surface with the presence of silanol groups and sodium cations that compensated the charge of the structural aluminum. In this case, AMX adsorption is done with calcined Al-MCM-41, and there is no structural CTA.

Calcination at temperatures of 550 °C and higher led to extensive dehydroxylation of the Al-MCM-41 surface (Figure 7c). The silanol groups were eliminated by condensation of the silica, with increased density and reduced hydrophobicity of the aluminosilicate surface.^43,44^

**Figure 8:**
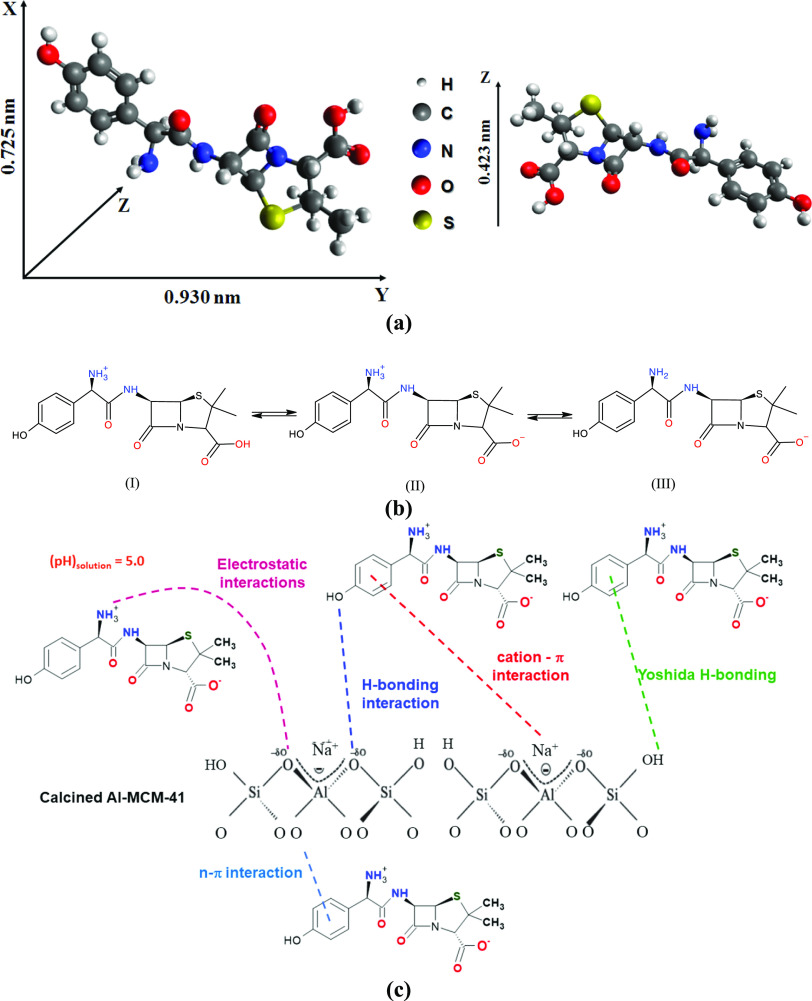
3D dimensions of the AMX molecule (a), speciation (b), and interactions with the Al-MCM-41 surface (c).

The chemical structure of the AMX molecule is shown in Figure 8a. In its zwitterionic form, the dimensions of the molecule are calculated as 0.725 × 0.930 × 0.423 nm (Figure 8a), allowing rapid Knudsen diffusion through the mesoporous of the Al-MCM-41 (*d_p_* = 2.99 nm), with low steric hindrance.^45^ These characteristics were consistent with the kinetic analyses, where the best fit was obtained using the pseudo-first order model (Section 3.2). The results (Figure 6) showed that the AMX adsorption capacity increased in the following order: Si/Al = 5 (SSA = 129 m^2^ g^–1^) < Si/Al = 16.1 (SSA = 840 m^2^ g^–1^) < Si/Al = 10.5 (SSA = 737 m^2^ g^–1^).

For adsorption at pH 5.0, species (II), shown in Figure 8b, could be considered most abundant (∼98.0%) in the medium.^46^ Under these conditions, the interactions between AMX and the Al-MCM-41 (Figure 8c) could include hydrogen bonding, π-π stacking, dipole–dipole hydrogen bonding interactions, Yoshida H-bonding, cation-π interactions, and n−π interactions.^47−50^ The adsorbent with the highest aluminum content (S-2) would provide the most effective cation-π interactions, which would favor the adsorption of AMX, but this would not be supported by the low specific area.

The lowest aluminum content of sample S-8 provided a larger adsorption area, but there were fewer adsorption sites, resulting in adsorption close to that obtained for sample S-2. With an increase in the aluminum content, the quantity of silanol groups on the surface decreased, while the number of Na^+^ cations increased. Hence, it is likely that the cation−π interactions increased with increase of the aluminum content, while the interactions involving silanols (Yoshida H-bonding and dipole–dipole hydrogen bridges) decreased. Therefore, effective removal of AMX from the medium required a balance between the available surface area and the viable intermolecular interactions for the adsorption to occur. A more detailed study of this adsorbent/adsorbate pair is needed for better understanding of the roles of the different parameters, including molecular interactions and the textural characteristics of the adsorbent.

## 4 Conclusions

The factorial design study of AMX adsorption on Al-MCM-41 revealed that only the Si/Al ratio had a statistically significant influence on the removal percentage and uptake capacity, while the effects of the synthesis time and temperature were not significant. The incorporation of high Al contents in the MCM-41 structure resulted in distortions of the mesophase framework due to the larger atomic radius of Al, compared to Si, which led to the generation of adsorbents with low specific surface area. However, the acid strength of the Al species, which were mostly present on the mesopore surface, significantly contributed to AMX adsorption. It is possible that the cation−π interactions increased with an increase in the aluminum content, while the interactions involving silanols (Yoshida H-bonding and dipole–dipole hydrogen bridges) decreased. The pseudo-first order kinetics and Freundlich isotherm suggested the formation of a complex heterogeneous adsorption surface due to the mesopore disorder resulting from the presence of aluminum species in the hexagonal structure. The excellent uptake performance (132 mg AMX g^–1^) of the adsorbent obtained using the optimized Si/Al ratio (10.5) indicated that Al-MCM-41 is a promising adsorbent for wastewater decontamination.

## ABBREVIATIONS

WHO: World Health Organization
Al-MCM-41: mobil composition of matter mesoporous silica with aluminum
AMX: amoxicillin
DDDs: defined daily doses
AMR: antibiotic and antimicrobial resistance
ECDC: European Centre for Disease Prevention and Control
*k*: factors in the factorial design
CP: central points in the factorial design
CTMABr: cetyltrimethylammonium bromide
TEOS: tetraethyl orthosilicate
NaOH: sodium hydroxide
NaAlO_2_: sodium aluminate
*C*_0_ and *C_e_*: initial and equilibrium concentrations of AMX in the liquid phase (mg L^–1^)
*V*: volume of AMX solution (mL)
*W*: mass of adsorbent (g of Al-MCM-41)
S-1 to S-8: samples synthesized in runs 1 to 8
S-CP: sample synthesized in the central point experiments (runs 9, 10, and 11)
SSA: specific surface area (m^2^ g^–1^)
q_t_ and q_e_: adsorption capacity of the adsorbent at time *t* and at equilibrium
*k*_1_: pseudo-first order rate constant
*K_F_* and *n*: Freundlich constants
*R*^2^: determination coefficient
